# Interpretable machine learning for cardiovascular disease risk prediction in cancer survivors: development and internal validation

**DOI:** 10.1186/s40959-026-00547-2

**Published:** 2026-07-20

**Authors:** Guoxing Zhang, Xueying Zhang, Chunyi Jia, Huanyu Zhou, Xiaojia Sun, Haifeng Liu

**Affiliations:** 1 ICU, Jilin Cancer Hospital, Changchun City, Jilin Province China; 2 Department of Medical Oncology, Jilin Cancer Hospital, Changchun City, Jilin Province China; 3 Thoracic Tumor Surgery, Jilin Cancer Hospital, Changchun City, Jilin Province China; 4 Oncology and Cardiology Department, Jilin Cancer Hospital, 1066 Jinhu Road, Chaoyang District, Changchun City, Jilin Province China

**Keywords:** Cancer survivors, Cardiovascular events, Machine learning, Risk prediction model

## Abstract

**Background:**

Cardiovascular disease (CVD) is a major concern among cancer survivors. However, the intersection of cancer and CVD has only recently gained broader attention, and substantial evidence gaps remain. This study aimed to identify risk factors associated with incident CVD in cancer survivors and to develop a machine learning model for CVD risk prediction.

**Methods:**

In this retrospective study, we included 2,500 patients receiving systemic antitumor therapy at Jilin Cancer Hospital; 188 incident CVD events were observed. Variables spanning demographic, clinical, tumor- and treatment-related, and laboratory domains were collected. The dataset was randomly split into a training set (75%) and an internal validation set (25%). Feature selection was performed using LASSO regression. Six prediction models were developed, including five machine learning algorithms (GBM, CoxBoost, XGBoost, SVM, and RSF) and a traditional Cox proportional hazards model. Model performance was evaluated using time-dependent receiver operating characteristic curves (AUC), calibration analyses, and decision curve analysis. Model interpretability was assessed using SHapley Additive exPlanations (SHAP).

**Results:**

In univariate analyses, 48 variables were associated with CVD risk (*P* < 0.20). LASSO regression identified 19 predictors for model development. Key predictors included elevated systolic blood pressure, specific cancer types, anthracycline use, and a history of hypertension. The XGBoost model demonstrated the best predictive performance, with an average AUC of 0.666, sensitivity of 69.8%, specificity of 60.7%, and overall accuracy of 61.4% in the internal validation set. The model showed good calibration and yielded a positive net benefit across a range of clinical thresholds. SHAP analysis indicated that cancer type, lack of anti-HER2 therapy, elevated systolic blood pressure, advanced T stage, advanced TNM stage, and higher uric acid levels were the most influential predictors.

**Conclusion:**

This study developed and internally validated interpretable machine learning models to predict CVD risk among cancer survivors. The models demonstrated good discrimination and calibration and outperformed traditional methods. By enabling individualized risk quantification and providing transparent interpretation of key predictors, this approach offers a practical tool to support personalized surveillance and prevention strategies in cardio-oncology.

**Supplementary Information:**

The online version contains supplementary material available at https://doi.org/10.1186/s40959-026-00547-2.

## Background

Advances in oncology have significantly improved cancer survival rates [1, 2], shifting clinical attention toward the management of treatment-related complications and long-term health outcomes among cancer survivors [1, 3]. Cardiovascular disease (CVD) has emerged as a leading cause of morbidity and mortality in this population. CVD risk is influenced by cancer type and stage, specific anticancer therapies (including drug class and cumulative dose), pre-existing comorbidities, and the accumulation of traditional CVD risk factors during or after treatment [4–6]. Studies indicate that patients with cancer face increased risks of major cardiovascular events, bleeding, and both cardiac and noncardiac mortality [7–10]. Notably, acute coronary syndrome (ACS) is being diagnosed more frequently in patients with cancer, who now account for approximately 3% of large-scale ACS registries [8]. The reported incidence of treatment-related cardiac ischemia ranges from 0.6% to 5%, depending on the therapeutic exposure [6, 11]. Consequently, integrating CVD risk assessment into cancer care through personalized surveillance, accurate prediction models, and timely preventive strategies is essential to improve long-term outcomes.

In response, risk prediction tools have attracted growing interest for individualized prevention and monitoring. The 2022 European Society of Cardiology (ESC) guidelines recommend the Heart Failure Association-International Cardio-Oncology Society (HFA-ICOS) risk assessment tool for patients scheduled to receive potentially cardiotoxic therapies [6, 12]. However, this tool is largely based on expert consensus and provides a qualitative checklist rather than a validated quantitative prediction model. Similarly, the American Society of Clinical Oncology (ASCO) guidelines propose a risk-stratification framework based on treatment type, age, and CVD risk factors, but do not include an integrated scoring system [13]. Although several attempts have been made to apply existing CVD risk prediction models, such as the Framingham risk score, to cancer survivors, their performance has been suboptimal [14]. A recent systematic review [15] highlighted that most existing predictive models have been developed and validated primarily in breast cancer populations [16–18] or childhood cancer survivors [19–21], with limited generalizability to other oncology cohorts. Moreover, many current tools categorize patients into broad risk groups rather than providing individualized risk estimates, which limits their ability to identify high-risk individuals who may benefit from targeted interventions.

Therefore, a robust, generalizable, and individualized prediction model for CVD risk in cancer survivors remains critically needed. This study aimed to identify independent risk factors associated with CVD and to clarify associations between different anticancer therapeutic regimens and specific cardiotoxicity phenotypes among cancer survivors who received systemic anticancer therapy. Building on these findings, we then developed and validated a machine-learning–based model to predict CVD risk in this population.

## Methods

### Study population and data source

This retrospective study used data from the electronic medical record system of Jilin Cancer Hospital. Cancer survivors were defined as patients who had survived for more than 3 years after their initial cancer diagnosis and had been followed for at least 2 years. Between December 2012 and July 2023, 5000 cancer survivors were enrolled. Inclusion criteria were as follows: (1) available and accurate contact information; (2) age 18–80 years; (3) receipt of systemic antitumor therapy; and (4) agreement to follow-up, with follow-up duration exceeding 30 days. Patients with pre-existing CVD or incomplete baseline cardiac assessments were excluded. The final analysis included 2500 participants, of whom 188 (7.5%) developed incident CVD. A flowchart of participant selection is shown in Supplementary Figure 1.

Patients were randomly allocated in a 3:1 ratio, with 75% assigned to the training set and 25% reserved for internal validation. The study protocol was approved by the Institutional Review Board of Jilin Province Cancer Hospital (approval number: 202603-009-01) and adhered to the principles outlined in the Declaration of Helsinki. Written informed consent was waived due to the retrospective nature of the study. Patient data were anonymized and kept confidential in accordance with ethical guidelines.

### Outcomes

The primary outcome was the first occurrence of a major adverse cardiovascular event after cancer therapy. CVD events included heart failure, cardiomyopathy, arterial or venous thrombosis, myocarditis, or clinically significant arrhythmia. The date of the first qualifying CVD event was defined as the date of the initial clinical diagnosis. Follow-up ended on the date of the first CVD event, death, loss to follow-up, or the end of the study period.

### Covariates

Predictors were collected across three domains: demographic characteristics, health status, and tumor- and treatment-related factors. These included age, sex (male or female), body mass index (BMI), cancer type, TNM stage, age at diagnosis, treatment details (agent, dose, and number of cycles), baseline cardiovascular status (left ventricular ejection fraction [LVEF], cardiac troponin I [cTnI], N‑terminal pro‑B‑type natriuretic peptide [NT‑proBNP]), comorbidities (hypertension, diabetes, and dyslipidemia), serial cardiac function assessments, and cardiovascular medication use.

### Pre-processing of machine learning features

The dataset initially contained 19 features: 9 continuous and 10 categorical. Continuous variables were Winsorized, with values capped at the 1 st and 99th percentiles. Missing values (<5% for key variables) were handled using multiple imputation. All features were subsequently standardized to a mean of 0 and a standard deviation of 1 to ensure comparable scaling across variables during model training.

### Statistical analysis

Categorical variables are presented as frequency (%), and continuous variables as mean ± standard deviation (for normally distributed data) or median (interquartile range). Associations between predictors and incident CVD were assessed using Cox proportional hazards regression, with results reported as hazard ratios (HRs) and 95% confidence intervals (CIs).

For predictive modeling, patients were randomly split into training (70%) and internal validation (30%) sets. Feature selection was performed using the least absolute shrinkage and selection operator (LASSO) regression in the training set, retaining variables with nonzero coefficients. Multiple predictive algorithms were trained and optimized using 10-fold cross-validation, including a Cox proportional hazards model (as a traditional benchmark), gradient boosting machine (GBM), CoxBoost, extreme gradient boosting (XGBoost), support vector machine for survival analysis (survival SVM), and random survival forest (RSF). Model performance was evaluated in the internal validation set using the time-dependent area under the receiver operating characteristic curve (AUC), Harrell’s C-index, calibration plots with the Brier score, and decision curve analysis (DCA). An ensemble model was constructed by averaging predictions from the top-performing individual models, with weights optimized on a held-out validation subset of the training data.

Model interpretability was assessed using SHapley Additive exPlanations (SHAP), which quantifies global feature importance and explains individual predictions. To evaluate time-specific predictive performance, separate models were developed for 1‑, 2‑, and 3‑year follow‑up intervals, in addition to an overall model using the full follow-up period. Patients were stratified into low-, intermediate-, and high-risk groups based on tertiles of predicted risk from the optimal model. Kaplan–Meier curves were plotted, and between-group differences were assessed using the log-rank test. Subgroup analyses were performed by tumor type and treatment regimen. Sensitivity analyses included complete-case analyses and varying the multiple imputation parameters.

All analyses were conducted in R version 4.3.0, and a two-sided *P* < 0.05 was considered statistically significant.

## Results

### Characteristics of the study population

A total of 2500 patients were included in the analysis. Baseline demographic, clinical, and treatment-related characteristics stratified by CVD occurrence are presented in Table 1. Compared with the non-CVD group (n=2312), patients who developed CVD (n=188) were significantly older (median age, 57.00 vs 54.00 years, *P* = 0.002), had a higher proportion of men (39.60% vs. 25.69%, *P* < 0.001), and had a lower prevalence of baseline hypertension (81.91% vs 89.36%, *P* = 0.003). Significant differences were also observed in key treatment modalities: the CVD group had greater use of anthracyclines (77.13% vs. 64.84%, *P* = 0.001) but lower use of fluoropyrimidines (61.70% vs. 74.83%, *P* < 0.001) and platinum-based agents (35.11% vs. 53.72%, *P* < 0.001). Among laboratory parameters, creatinine (61.75 vs 58.90 μmol/L; *P* = 0.016), uric acid (298.30 vs. 279.35 μmol/L, *P* = 0.005), systolic blood pressure (125.00 vs. 120.00 mmHg, *P* < 0.001), and eosinophil count (0.10 vs. 0.08 10^9^/L, *P* < 0.001) were significantly higher in the CVD group.

**Table 1:** Characteristics of the study population

Variable	Overall (*n*=2500)	non-CVD (*n*=2312)	CVD (*n*=188)	*P*
Age, years	54.00 [47.00, 61.00]	54.00 [47.00, 61.00]	57.00 [50.00, 63.00]	0.002
Gender, (%)				<0.001
Female	1832 (73.28)	1718 (74.31)	114 (60.64)	
Male	668 (26.72)	594 (25.69)	74 (39.36)	
Hypertension, (%)				0.003
No	2220 (88.80)	2066 (89.36)	154 (81.91)	
Yes	280 (11.20)	246 (10.64)	34 (18.09)	
Diastolic BP, mmHg	80.00 [76.00, 82.00]	80.00 [76.00, 81.25]	80.00 [76.00, 83.00]	0.301
Systolic BP, mmHg	120.00 [120.00, 130.00]	120.00 [120.00, 130.00]	125.00 [120.00, 139.25]	<0.001
Diabetes, (%)				0.327
No	2360 (94.40)	2186 (94.55)	174 (92.55)	
Yes	140 (5.60)	126 (5.45)	14 (7.45)	
Lung cancer, (%)				
No	1451 (58.04)	1374 (59.43)	77 (40.96)	<0.001
Yes	1049 (41.96)	938 (40.57)	111 (59.04)	
Breast cancer, (%)				
No	1299 (51.96)	1174 (50.78)	125 (66.49)	<0.001
Yes	1201 (48.04)	1138 (49.22)	63 (33.51)	
Liver cancer, (%)				0.656
No	2427 (97.08)	2243 (97.02)	184 (97.87)	
Yes	73 (2.92)	69 (2.98)	4 (2.13)	
Gastric cancer, (%)				0.406
No	2323 (92.92)	2145 (92.78)	178 (94.68)	
Yes	177 (7.08)	167 (7.22)	10 (5.32)	
T stage, (%)				0.32
T0	125 (5.00)	110 (4.76)	15 (7.98)	
T1	689 (27.56)	643 (27.81)	46 (24.47)	
T2	1021 (40.84)	945 (40.87)	76 (40.43)	
T3	287 (11.48)	267 (11.55)	20 (10.64)	
T4	378 (15.12)	347 (15.01)	31 (16.49)	
N stage, (%)				0.868
N0	1020 (40.80)	943 (40.79)	77 (40.96)	
N1	580 (23.20)	536 (23.18)	44 (23.40)	
N2	554 (22.16)	516 (22.32)	38 (20.21)	
N3	346 (13.84)	317 (13.71)	29 (15.43)	
M stage, (%)				0.552
M0	1822 (72.88)	1681 (72.71)	141 (75.00)	
M1	678 (27.12)	631 (27.29)	47 (25.00)	
TNM_stage, (%)				0.274
Ⅰ	559 (22.36)	510 (22.06)	49 (26.06)	
Ⅱ	567 (22.68)	533 (23.05)	34 (18.09)	
Ⅲ	718 (28.72)	659 (28.50)	59 (31.38)	
Ⅳ	656 (26.24)	610 (26.38)	46 (24.47)	
ALT, U/L	17.70 [13.50, 24.90]	17.65 [13.50, 24.70]	18.35 [13.35, 25.83]	0.450
AST, U/L	19.70 [16.70, 24.10]	19.70 [16.70, 24.10]	19.35 [16.08, 23.60]	0.370
GGT, U/L	23.40 [16.80, 37.50]	23.30 [16.70, 37.23]	25.30 [17.15, 41.23]	0.219
TBIL, μmol/L	12.10 [9.40, 15.60]	12.20 [9.40, 15.60]	11.90 [9.50, 14.93]	0.794
DBIL, μmol/L	3.00 [2.30, 4.20]	3.00 [2.30, 4.20]	3.10 [2.20, 4.40]	0.967
IBIL, μmol/L	9.00 [6.60, 11.90]	9.00 [6.60, 11.90]	8.80 [6.57, 11.75]	0.741
ALB, g/L	43.10 [40.70, 45.10]	43.10 [40.70, 45.10]	43.00 [40.38, 44.90]	0.532
GLO, g/L	29.40 [26.80, 32.40]	29.40 [26.80, 32.30]	29.05 [26.20, 32.52]	0.253
TP, g/L	72.70 [69.00, 76.10]	72.71 [69.00, 76.10]	72.20 [68.60, 76.12]	0.802
ALB/GLO ratio	1.46 [1.32, 1.61]	1.46 [1.32, 1.61]	1.45 [1.32, 1.65]	0.487
TBA, μmol/L	2.68 [1.60, 4.60]	2.60 [1.60, 4.60]	2.80 [1.60, 4.32]	0.979
ALP, U/L	72.30 [58.80, 89.90]	72.20 [58.58, 89.73]	73.10 [60.80, 91.23]	0.508
ChE, U/L	8042.00 [6842.75, 9331.00]	8047.00 [6854.75, 9317.35]	7980.00 [6677.25, 9356.00]	0.805
Cr, μmol/L	59.00 [52.00, 67.93]	58.90 [51.90, 67.80]	61.75 [53.50, 70.50]	0.016
Urea, mmol/L	4.60 [3.70, 5.60]	4.60 [3.70, 5.60]	4.80 [3.70, 5.62]	0.667
UA, μmol/L	280.45 [232.80, 336.72]	279.35 [232.30, 334.35]	298.30 [246.70, 362.05]	0.005
Cys-C, mg/L	0.88 [0.78, 0.99]	0.88 [0.78, 0.99]	0.90 [0.81, 1.03]	0.086
Glucose, mmol/L	5.41 [5.05, 5.93]	5.41 [5.04, 5.92]	5.46 [5.15, 5.98]	0.107
K, mmol/L	4.09 [3.88, 4.31]	4.09 [3.88, 4.31]	4.05 [3.81, 4.30]	0.128
Na, mmol/L	139.60 [138.12, 140.90]	139.60 [138.10, 140.90]	139.70 [138.28, 141.33]	0.182
Cl, mmol/L	104.20 [102.20, 106.10]	104.20 [102.20, 106.10]	104.30 [102.07, 106.00]	0.866
Ca, mmol/L	2.36 [2.29, 2.42]	2.36 [2.29, 2.42]	2.36 [2.29, 2.42]	0.718
P, mmol/L	1.17 [1.06, 1.29]	1.17 [1.06, 1.29]	1.17 [1.04, 1.29]	0.802
Mg, mmol/L	0.86 [0.82, 0.90]	0.86 [0.82, 0.90]	0.86 [0.81, 0.91]	0.985
TT, s	14.33 [13.60, 15.10]	14.33 [13.60, 15.10]	14.35 [13.50, 15.00]	0.463
PT, s	11.18 [10.70, 11.70]	11.18 [10.70, 11.70]	11.13 [10.60, 11.70]	0.743
APTT, s	31.10 [29.10, 33.10]	31.10 [29.10, 33.10]	31.25 [29.28, 33.10]	0.317
PT-INR	1.02 [0.97, 1.06]	1.02 [0.97, 1.06]	1.02 [0.96, 1.08]	0.872
Fibrinogen, g/L	3.18 [2.74, 3.73]	3.16 [2.74, 3.70]	3.36 [2.73, 3.82]	0.065
PTA, %	97.00 [91.00, 104.00]	97.00 [91.00, 104.00]	97.00 [88.00, 106.00]	0.784
WBC, 10^9^/L	6.10 [5.10, 7.30]	6.10 [5.00, 7.30]	6.30 [5.30, 7.82]	0.064
RBC, 10^12^/L	4.54 [4.28, 4.82]	4.54 [4.28, 4.81]	4.58 [4.29, 4.84]	0.188
MCV, fL	91.20 [88.00, 94.23]	91.10 [88.00, 94.20]	91.50 [88.07, 95.10]	0.349
MCHC, g/L	331.00 [324.00, 337.00]	331.00 [324.00, 337.00]	330.00 [323.00, 336.00]	0.632
MCH, pg	30.20 [29.00, 31.30]	30.20 [29.00, 31.30]	30.30 [29.00, 31.60]	0.632
RDW-CV, %	13.20 [12.80, 13.80]	13.20 [12.70, 13.80]	13.30 [12.80, 13.80]	0.277
Lymph%, %	27.80 [22.26, 33.56]	27.83 [22.26, 33.54]	27.51 [22.44, 34.12]	0.035
Lymph, 10^9^/L	1.65 [1.32, 2.05]	1.65 [1.31, 2.04]	1.75 [1.37, 2.16]	0.035
Mono%, %	6.36 [5.31, 7.60]	6.35 [5.31, 7.59]	6.48 [5.41, 7.67]	0.035
Mono, 10^9^/L	0.38 [0.31, 0.48]	0.38 [0.31, 0.48]	0.41 [0.32, 0.50]	0.035
Neut%, %	63.28 [56.77, 69.27]	63.25 [56.80, 69.30]	63.47 [56.51, 68.95]	0.211
Neut, 10^9^/L	3.81 [2.98, 4.85]	3.80 [2.97, 4.84]	3.90 [3.11, 5.06]	0.211
Hct, %	0.41 [0.39, 0.44]	0.41 [0.39, 0.44]	0.42 [0.39, 0.45]	0.077
Eosin%, %	1.31 [0.73, 2.28]	1.31 [0.72, 2.25]	1.55 [0.86, 2.83]	<0.001
Baso%, %	0.48 [0.36, 0.64]	0.48 [0.36, 0.64]	0.49 [0.36, 0.67]	0.089
Eosin, 10^9^/L	0.08 [0.04, 0.14]	0.08 [0.04, 0.14]	0.10 [0.05, 0.18]	<0.001
Baso, 10^9^/L	0.03 [0.02, 0.04]	0.03 [0.02, 0.04]	0.03 [0.02, 0.04]	0.089
Hb, g/L	136.80 [128.80, 145.30]	136.75 [128.67, 145.02]	137.45 [129.68, 147.12]	0.198
PLT, 10^9^/L	234.00 [198.00, 280.00]	234.00 [198.00, 280.00]	232.00 [200.00, 285.25]	0.65
MPV, fL	8.20 [7.60, 8.90]	8.30 [7.60, 8.90]	8.00 [7.50, 8.80]	0.01
PDW, fL	16.50 [16.20, 16.90]	16.50 [16.20, 16.90]	16.50 [16.20, 16.90]	0.684
Chemotherapy, (%)				0.036
No	299 (11.96)	286 (12.37)	13 (6.91)	
Yes	2201 (88.04)	2026 (87.63)	175 (93.09)	
Anthracycline, (%)				0.001
No	1644 (65.76)	1499 (64.84)	145 (77.13)	
Yes	856 (34.24)	813 (35.16)	43 (22.87)	
Fluoropyrimidine, (%)				<0.001
No	1846 (73.84)	1730 (74.83)	116 (61.70)	
Yes	654 (26.16)	582 (25.17)	72 (38.30)	
Platin, (%)				<0.001
No	1308 (52.32)	1242 (53.72)	66 (35.11)	
Yes	1192 (47.68)	1070 (46.28)	122 (64.89)	
Taxane, (%)				0.618
No	1114 (44.56)	1034 (44.72)	80 (42.55)	
Yes	1386 (55.44)	1278 (55.28)	108 (57.45)	
Immunosuppressant, (%)				<0.001
No	1588 (63.52)	1445 (62.50)	143 (76.06)	
Yes	912 (36.48)	867 (37.50)	45 (23.94)	
Other chemotherapy, (%)				0.05
No	1981 (79.24)	1843 (79.71)	138 (73.40)	
Yes	519 (20.76)	469 (20.29)	50 (26.60)	
Targeted therapy, (%)				0.421
No	1696 (67.84)	1563 (67.60)	133 (70.74)	
Yes	804 (32.16)	749 (32.40)	55 (29.26)	
Anti-HER2, (%)				0.642
No	2239 (89.56)	2073 (89.66)	166 (88.30)	
Yes	261 (10.44)	239 (10.34)	22 (11.70)	
Other targeted therapy, (%)				0.163
No	2390 (95.60)	2206 (95.42)	184 (97.87)	
Yes	110 (4.40)	106 (4.58)	4 (2.13)	
Antiangiogenic-TKI, (%)				0.271
No	2264 (90.56)	2089 (90.35)	175 (93.09)	
Yes	236 (9.44)	223 (9.65)	13 (6.91)	
EGFR-TKI, (%)				0.487
No	2145 (85.80)	1980 (85.64)	165 (87.77)	
Yes	355 (14.20)	332 (14.36)	23 (12.23)	
Immunotherapy, (%)				0.279
No	2245 (89.80)	2081 (90.01)	164 (87.23)	
Yes	255 (10.20)	231 (9.99)	24 (12.77)	
Immune checkpoint inhibitor, (%)				0.279
No	2245 (89.80)	2081 (90.01)	164 (87.23)	
Yes	255 (10.20)	231 (9.99)	24 (12.77)	
Radiotherapy, (%)				0.203
No	1493 (59.72)	1372 (59.34)	121 (64.36)	
Yes	1007 (40.28)	940 (40.66)	67 (35.64)	
Chest radiotherapy, (%)				0.004
No	1971 (78.84)	1839 (79.54)	132 (70.21)	
Yes	529 (21.16)	473 (20.46)	56 (29.79)	

### Risk factors associated with CVD incidence

**Fig. 1:**
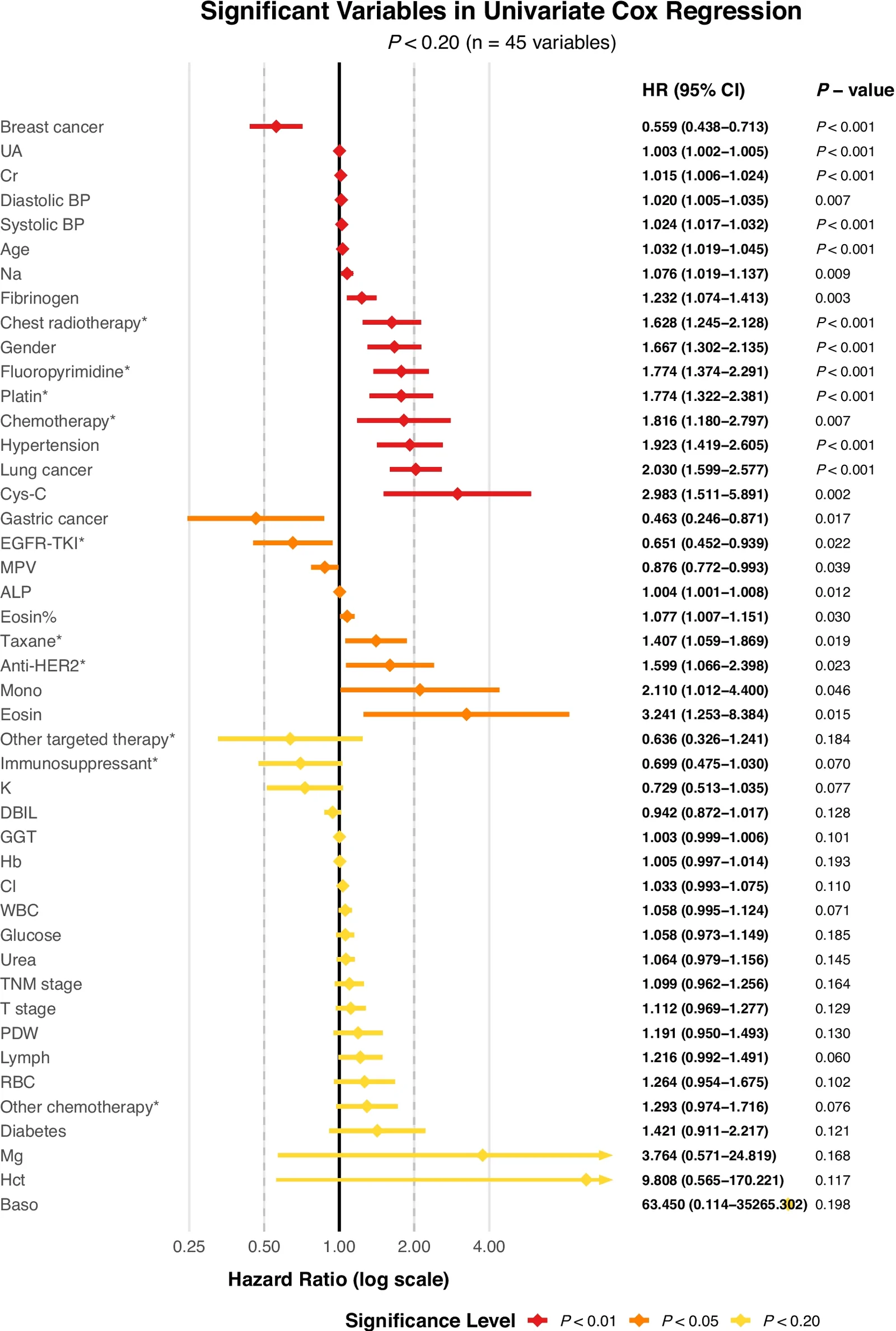
Significance of the univariable association between risk factors and CVD incidence

The results of the univariable Cox proportional hazards regression analyses are shown in Figure 1. A total of 48 variables met the prescreening threshold (*P* < 0.20). The most statistically significant factors (*P* < 0.01) included Cys-C (HR=2.98, 95% CI: 1.51, 5.89), followed by lung cancer (HR=2.03, 95% CI: 1.60, 2.58), hypertension (HR=1.92, 95% CI: 1.42, 2.61), gender (HR=1.67, 95% CI: 1.30, 2.14), chemotherapy (HR=1.82, 95% CI: 1.80, 2.80), platin use (HR=1.77, 95% CI: 1.32, 2.38), use of fluoropyrimidine chemotherapy (HR=1.77, 95% CI: 1.37, 2.29), chest radiotherapy (HR=1.63, 95% CI: 1.25, 2.13), age (HR=1.03, 95% CI: 1.02, 1.05), systolic blood pressure (HR=1.02, 95% CI: 1.02, 1.03), diastolic blood pressure (HR=1.02, 95% CI: 1.01, 1.04), Crea (HR=1.02, 95% CI: 1.01, 1.02), uric acid level (HR=1.00, 95% CI: 1.00, 1.01), and breast cancer (HR=0.56, 95% CI: 0.44, 0.71). Other predictors with *P*< 0.05 included ALP, eosinophil count, gastric cancer, taxane, eosinophil percent, MPV, Mono, EGFR-TKI, and Anti-HER2. Several variables showed marginal associations (0.05 < *P* < 0.20), including other targeted therapy, immunosuppressant, K, DBIL, GGT, Hb, CIM, WBC, glucose, urea, TNM stage, T stage, PDW, lymph, RBC, other chemotherapy, diabetes, Mg, Hct, and Baso. The full univariable analysis results for all variables are provided in Supplementary Figure 2.

The correlation matrix of the analyzed features is shown in Supplementary Figure** 3**, indicating several moderate to high correlations among the variables.

### Development and validation of the CVD prediction model

**Fig. 2:**
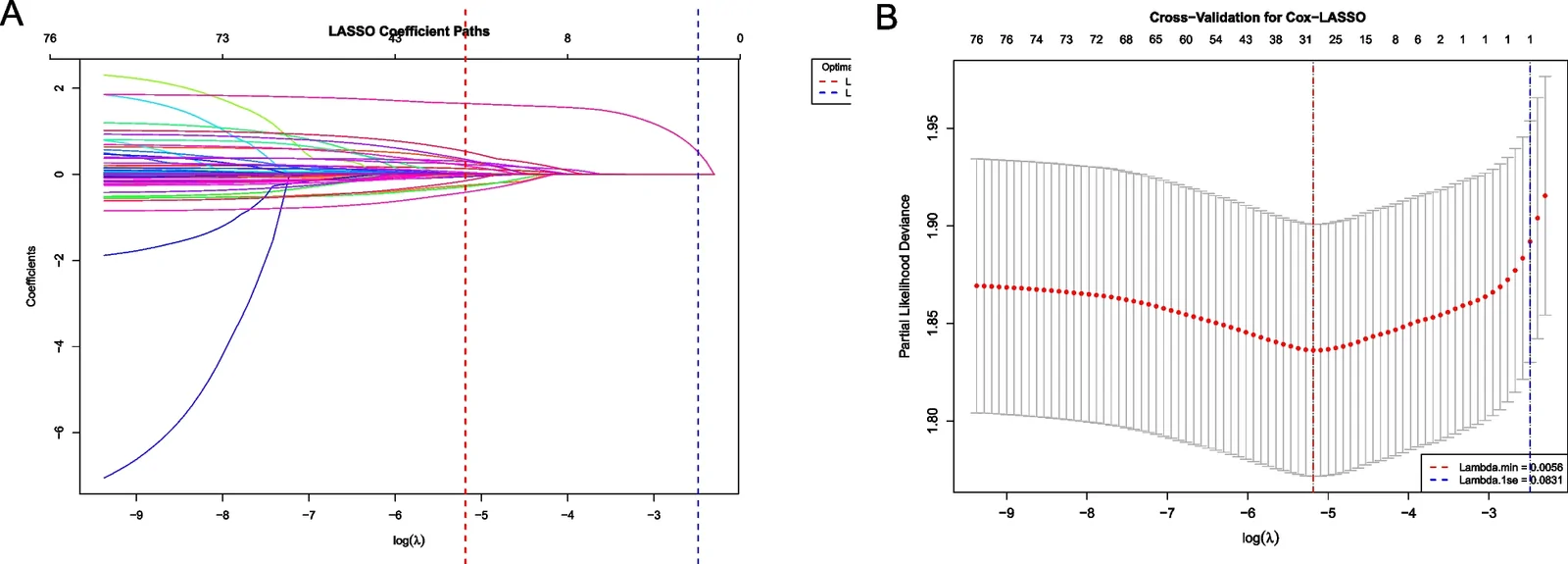
LASSO Coefficient Profiles and Cross-Validation Error Plot. **A** LASSO coefficient profiles of the 48 prescreened CVD-associated variables (*P*<0.20 in univariable analysis). Each curve represents the change in regression coefficient of a single variable with the variation of the log-transformed tuning parameter (λ). **B** Cross-validation error plot for optimal λ selection in LASSO regression. The optimal λ was determined via 10-fold cross-validation based on the minimum partial likelihood deviance, and variables with non-zero coefficients at this optimal λ were retained for subsequent predictive model construction

Before model development, the cohort was randomly split into training (n=1750) and validation (n=750) sets. Baseline characteristics were comparable between the training and validation sets, with no significant differences. Feature selection using LASSO regression identified 19 predictors for model construction, including cancer type, anti-HER2, systolic blood pressure, T stage, TNM stage, uric acid level, Lymph, K, anthracycline use, FBG, NA, Cys-C, MPV, chest radiotherapy, use of fluoropyrimidine chemotherapy, taxane, gender, platin, and hypertension. The results are shown in Figure 2.

**Fig. 3:**
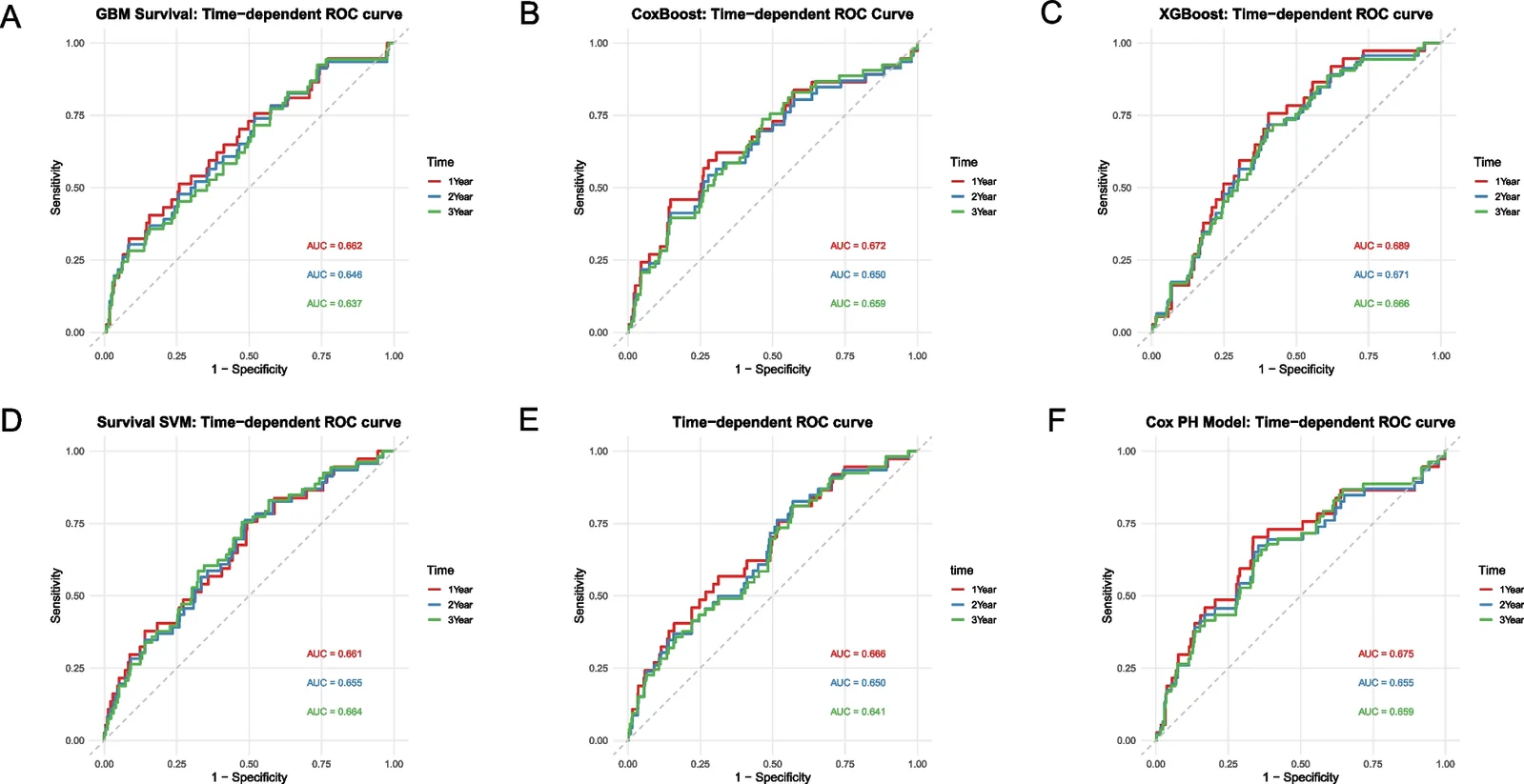
Receiver Operating Characteristic Curves of Different Models. Time-dependent receiver operating characteristic (ROC) curves were plotted to evaluate the discriminative performance of each model for 1-year, 2-year, and 3-year CVD risk prediction in the internal validation set, with the area under the curve (AUC) value labeled for each time point. **A** Time-dependent ROC curves of the Gradient Boosting Machine (GBM) model. **B** Time-dependent ROC curves of the CoxBoost model. **C** Time-dependent ROC curves of the eXtreme Gradient Boosting (XGBoost) model. **D** Time-dependent ROC curves of the survival Support Vector Machine (survival SVM) model. **E** Time-dependent ROC curves of the Random Survival Forest (RSF) model. **F** Time-dependent ROC curves of the traditional Cox proportional hazards regression model.

The predictive performance of five machine learning models and a traditional Cox model was evaluated in the validation set. The results are presented in Table 2. Model discrimination, assessed using the AUC, is shown in Figure 3; higher AUC values indicate better predictive performance. The XGBoost model demonstrated the highest discriminative ability, with a 3-year AUC of 0.666, sensitivity of 69.8%, specificity of 60.7%, positive predictive value (PPV) of 12.3%, and overall accuracy of 61.4%. Performance was consistent across the 1-, 2-, and 3-year time points. In contrast, the Random Survival Forest (RSF) model performed worst, with a 3-year AUC of 0.62 and an accuracy of 45.5%. CoxBoost (3-year AUC, 0.659; accuracy, 55.0%), traditional Cox (3-year AUC 0.659, accuracy 62.2%), and SVM (3-year AUC 0.664, accuracy 54.1%) models showed intermediate performance. The GBM model had a 3-year AUC of 0.637 and an accuracy of 81.1%.

**Table 2:** Performance Metrics of Six Models in the Validation Set

Model	Time	AUC	Sensitivity	Specificity	PPV	NPV	Accuracy	F1_Score
gbm	1Year	0.662	0.514	0.742	0.094	0.967	0.73	0.159
	2Year	0.646	0.739	0.483	0.087	0.965	0.499	0.155
	3Year	0.637	0.358	0.846	0.156	0.943	0.811	0.217
	AVERAGE	0.648	0.537	0.690	0.112	0.958	0.68	0.177
coxboost	1Year	0.672	0.622	0.695	0.096	0.972	0.691	0.167
	2Year	0.65	0.543	0.723	0.115	0.96	0.711	0.19
	3Year	0.659	0.736	0.536	0.111	0.962	0.55	0.194
	AVERAGE	0.660	0.634	0.651	0.107	0.965	0.651	0.184
xgb	1Year	0.689	0.757	0.596	0.089	0.979	0.604	0.16
	2Year	0.671	0.717	0.6	0.106	0.97	0.607	0.185
	3Year	0.666	0.698	0.607	0.123	0.962	0.614	0.21
	AVERAGE	0.675	0.724	0.601	0.106	0.970	0.608	0.185
ssvm	1Year	0.661	0.757	0.507	0.074	0.976	0.519	0.135
	2Year	0.655	0.761	0.514	0.094	0.97	0.53	0.168
	3Year	0.664	0.755	0.524	0.111	0.964	0.541	0.194
	AVERAGE	0.66	0.758	0.515	0.093	0.97	0.53	0.166
rsf	1Year	0.638	0.568	0.684	0.086	0.968	0.678	0.149
	2Year	0.625	0.783	0.426	0.083	0.967	0.449	0.15
	3Year	0.62	0.774	0.43	0.097	0.96	0.455	0.172
	AVERAGE	0.628	0.708	0.513	0.089	0.965	0.527	0.157
cox	1Year	0.675	0.703	0.664	0.098	0.977	0.666	0.173
	2Year	0.655	0.674	0.646	0.112	0.968	0.648	0.193
	3Year	0.659	0.679	0.618	0.123	0.961	0.622	0.209
	AVERAGE	0.663	0.685	0.643	0.111	0.969	0.645	0.192

The XGBoost model showed good calibration across the range of predicted probabilities. The calibration curve indicated close agreement between predicted risks and observed event rates (Supplementary Figure 4). Decision curve analysis (Supplementary Figure 5) supported the clinical utility of the intervention, demonstrating a positive net benefit across a wide range of threshold probabilities for clinical intervention (approximately 1%–25%) and outperforming the “treat all” and “treat none” strategies. Kaplan–Meier analysis based on binary risk stratification from the XGBoost model (Supplementary Figure 6) showed significant separation in event-free survival between the high-risk and low-risk groups (log-rank *P* < 0.001), with the high-risk group experiencing a substantially higher and earlier incidence of CVD events.

### Model interpretation by SHAP analysis

**Fig. 4:**
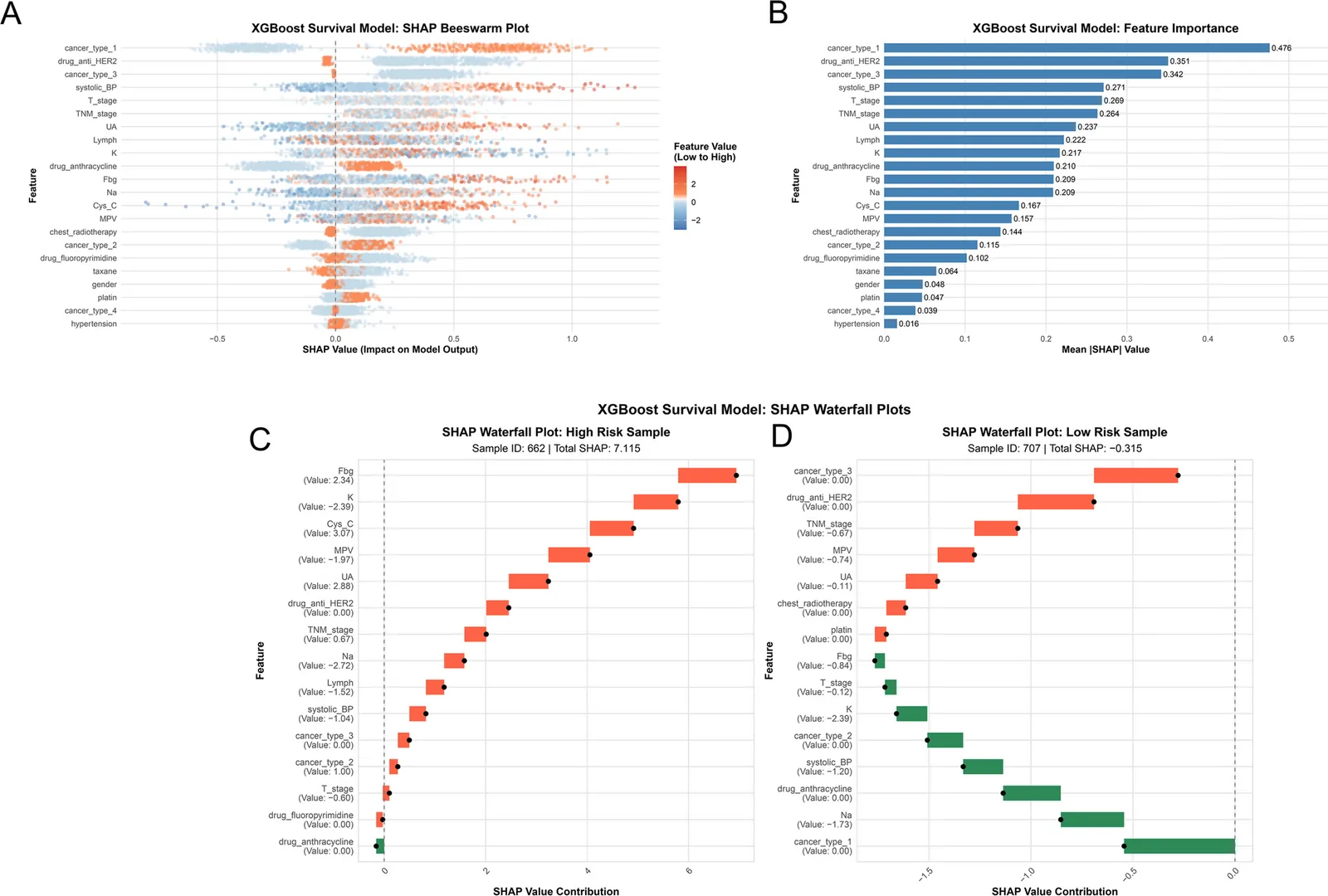
Model Interpretation by SHAP Analysis. SHAP analysis was performed to quantify the global feature importance of the optimal XGBoost model and explain the individualized CVD risk prediction for each patient. **A** SHAP summary plot ranking the top 15 predictive features by their mean absolute SHAP values, which reflects the overall magnitude of each variable's contribution to the model's CVD risk prediction. The color gradient represents the variable value from low to high. **B** SHAP waterfall plot for a representative high-risk patient, illustrating the additive contribution of each key feature to the shift of predicted CVD risk from the model's baseline value (base log-odds) to the final individual risk score. Red bars indicate features that increase CVD risk, while blue bars indicate features that reduce CVD risk. **C** SHAP dependence plot of systolic blood pressure (one of the top 3 predictors), demonstrating the nonlinear relationship between systolic blood pressure levels and its corresponding SHAP values, as well as the marginal effect of this variable on the predicted CVD risk. **D** Stratified SHAP feature contribution plot, displaying the average SHAP values of core predictors in low-, intermediate-, and high-CVD risk groups stratified by tertiles of the XGBoost model's predicted risk, to visualize the differential driving factors of CVD risk across different risk strata.

SHAP analysis was used to interpret predictions from the optimal XGBoost model (Figure 4). The summary plot (Figure 4A), which ranks the top 15 features by mean absolute SHAP value, identified the most influential predictors. Key factors contributing to a higher predicted CVD risk included non-lung cancer type, absence of Anti-HER2 therapy, higher systolic blood pressure, advanced T stage (e.g., T3/T4), advanced TNM stage (e.g., Stage III/IV), and higher uric acid levels. Female gender and receipt of fluoropyrimidine chemotherapy were associated with lower predicted CVD risk. The waterfall plot (Figure 4B) illustrates the individualized prediction for a representative high-risk patient, showing how each feature contributes additively to shift the model output from the base log-odds value to the final predicted risk. For this patient, a non-lung cancer type, higher systolic blood pressure, and advanced T stage were the primary drivers of the elevated risk score.

## Discussion

This study developed and validated a machine learning model to predict CVD risk in cancer survivors after systemic antitumor therapy. This model provides a data-driven tool for individualized risk quantification, complementing existing qualitative guidelines and supporting tailored surveillance strategies.

Our model demonstrated moderate discriminative performance (AUC ≈ 0.666). While not exceptionally high, this level is comparable to existing cardio-oncology tools, many of which are qualitative checklists without formal validation of discriminative performance. The decision curve analysis supported a positive net benefit across clinically relevant threshold probabilities. The moderate AUC likely reflects the complex, multifactorial nature of CVD in cancer survivors, and we view this tool as a decision-support aid to be used alongside clinical judgment. Our findings align with and extend current knowledge regarding CVD risk factors in cancer survivors. Confirmation of established risk factors, including hypertension (elevated systolic and diastolic blood pressure), male sex, older age, and impaired renal function (elevated serum creatinine and cystatin C), reinforces the biological plausibility of the model [13, 22, 23]. Moreover, the model captured distinct cardiotoxic risks associated with specific anticancer therapies, including anthracyclines, fluoropyrimidines, and chest radiotherapy [24–28], consistent with known mechanisms of myocardial injury, coronary vasospasm, and vascular damage, respectively [29–31]. The results align closely with key clinical risk elements emphasized in consensus tools, such as the HFA-ICOS checklist [6, 12]. Notably, the analysis also identified several significant predictors that extend beyond conventional risk assessment frameworks, including anti‑HER2 therapy and taxane-based chemotherapy, elevated inflammatory markers, and cancer-related prognostic indicators such as advanced T stage and specific cancer subtypes. Incorporation of these variables, spanning therapy‑specific CVD hazards [6, 32, 33], host inflammatory response in cancer‑related CVD [34], and tumor biology [34, 35], provides a more nuanced and multidimensional risk profile than currently available qualitative tools and may enable earlier identification of at-risk patients who could be overlooked by guideline-driven checklists alone.

The model directly addresses key methodological and clinical gaps highlighted in recent systematic reviews of CVD prediction tools. As synthesized by Gomes et al. [4], the current landscape of CVD risk prediction models for cancer survivors is characterized by a frequent lack of external validation, which limits generalizability beyond the original development cohorts [14, 16, 17]. This concern is reinforced by methodological studies emphasizing that rigorous external validation is essential before clinical adoption in other populations. Furthermore, many widely used tools, including the consensus-based HFA-ICOS checklist, provide qualitative risk categories (e.g., low, medium, high) rather than individualized quantitative risk estimates [6, 12]. This approach contrasts with the need for precise, personalized risk stratification in clinical decision-making. In addition, many established models were developed primarily using data from traditional chemotherapies and do not fully incorporate CVD risks associated with the broader spectrum of modern anticancer therapies [36]. Similarly, the review by Altena et al. [15] highlighted the urgent need for evidence-based, generalizable tools to predict CVD morbidity in the heterogeneous adult cancer population, moving beyond consensus-driven checklists. This study responds to these calls by developing and validating a model in a mixed-cancer cohort, thereby enhancing generalizability across oncology subtypes. Crucially, the model provides a quantitative, continuous 3-year risk score that enables more granular risk stratification than the binary or categorical outputs of many existing tools. The robust performance in both internal and external validation cohorts supports potential reliability across diverse clinical settings. By integrating a broad set of predictors spanning demographics, traditional CVD risk factors, specific anticancer drug exposures, and novel inflammatory biomarkers, this tool represents a practical and meaningful advance toward personalized risk assessment and preventive care in cardio-oncology.

The application of SHAP provided data-driven insights into the hierarchy of risk contributors, revealing a distinct pattern in which cancer-specific factors outweighed traditional CVD variables in predictive importance in this oncology cohort. Notably, SHAP values are expressed on the log-odds scale, and their magnitude reflects the marginal contribution of each feature to the model's prediction rather than directly quantifying effect size in clinically familiar units. Specifically, the SHAP summary plot identified cancer type as the most influential feature, followed by Anti-HER2 therapy, whereas established factors such as hypertension ranked third. This ordering suggests that overall risk is primarily defined by the biology of the primary malignancy and the specificity of its treatment, with conventional CVD risk factors acting as potent modifiers within that context. The strong contributions of T stage and TNM stage further support that advanced tumor burden is a key determinant of CVD risk, potentially mediated through systemic inflammation, hypercoagulability, and increased metabolic demand [37, 38]. Importantly, the prominent contribution of Anti-HER2 therapy illustrates the model's ability to quantify therapy-specific risk and to distinguish the reversible HER2-signaling blockade mechanism from other cardiotoxic pathways [6, 32, 33]. The inclusion of uric acid as a major contributor suggests a potential role of purine metabolism and oxidative stress in cancer-related CVD. This pathway is increasingly recognized in cardio-oncology literature [39, 40]. Further, a modest SHAP value does not necessarily indicate negligible clinical relevance, particularly when multiple features act additively to shift an individual's predicted risk. The clinical significance of these findings is best evaluated by their biological plausibility, their alignment with established mechanisms of cardiotoxicity, and their potential to inform actionable clinical decisions. This data-driven prioritization of oncological variables over purely cardiological factors represents a nuance that is not captured by current generalized risk stratification frameworks. Importantly, the use of SHAP for model interpretation aligns with a growing body of literature on explainable machine learning in oncology [41–43]. These studies collectively underscore a paradigm shift toward model transparency in clinical oncology, where interpretability is increasingly recognized as essential for translating machine learning innovations into actionable clinical tools.

This study has several strengths. It integrates multimodal clinical, treatment, and biomarker data with interpretable machine learning to develop a dynamic, time-dependent prediction model. Model performance was rigorously validated using independent multicenter data, supporting potential generalizability across diverse clinical settings. Several limitations should be acknowledged. First, despite the inclusion of a broad range of predictors, some potentially relevant factors were not available (e.g., physical activity, diet, smoking, socioeconomic status). This omission may result in residual confounding and limit the comprehensiveness of the model [44]. Second, our model was developed and internally validated using data from a single regional cancer center in Northeast China, which may limit its generalizability to other healthcare systems and ethnic groups. Although the training and validation splits were randomly allocated and baseline characteristics were well balanced, we acknowledge that the lack of an independent external cohort to test model performance remains a major constraint. Moreover, the single-center design may have introduced selection bias, potentially overrepresenting patients with more advanced disease or complex treatment histories. Regional variations in treatment practices, supportive care standards, and cardiovascular risk factors may also affect model performance. Future multicenter studies are needed to assess the transportability of our findings. Third, the pathway for clinical translation remains to be defined. Although the model may support risk stratification, prospective interventional studies are needed to determine whether acting on its predictions improves patient outcomes (e.g., through targeted monitoring or preventive therapy) and whether such an approach is cost-effective.

## Conclusions

This study developed and validated an interpretable machine-learning model to predict CVD risk in cancer survivors after systemic antitumor therapy. The XGBoost model demonstrated good discrimination and calibration, providing a quantitative, individualized risk estimate that can inform discussions regarding cardiotoxic treatment, patient surveillance, and preventive interventions. By elucidating the contributions of both oncological and traditional CVD risk factors, the model offers a practical tool to advance personalized risk assessment in cardio-oncology and provides a framework for developing more sophisticated, cancer-type-specific models in the future.

## Supplementary Information

Supplementary Material 1.

Supplementary Material 2.

Supplementary Material 3.

Supplementary Material 4.

Supplementary Material 5.

Supplementary Material 6.

## Data Availability

The datasets generated and analyzed during the current study are available from the corresponding author on reasonable request.

## Abbreviations

CVD: Cardiovascular disease
ACS: Acute coronary syndrome
ESC: European Society of Cardiology
HFA-ICOS: Heart Failure Association-International Cardio-Oncology Society
BMI: Body mass index
LVEF: Left ventricular ejection fraction
cTnI: Cardiac troponin I
NT‑proBNP: N‑terminal pro‑B‑type natriuretic peptide
HRs: Hazard ratios
Cis: Confidence intervals
LASSO: Least absolute shrinkage and selection operator
GBM: Gradient boosting machine
XGBoost: Extreme gradient boosting
RSF: Random survival forest
DCA: Decision curve analysis

## References

[1] Tan SY, Turner J, Kerin-Ayres K, Butler S, Deguchi C, Khatri S, et al. Health concerns of cancer survivors after primary anti-cancer treatment. Support Care Cancer. 2019;27:3739–47. 10.1007/s00520-019-04664-w 30710242

[2] Choi A, Kim S, Kim S, Cho I, Cha MJ, You SC. Atherosclerotic cardiovascular disease in cancer survivors: current evidence, risk prediction, prevention, and management. J Lipid Atheroscler. 2025;14:30–9. 10.12997/jla.2025.14.1.30 PMC11791415 39911963

[3] Geenen MM, Cardous-Ubbink MC, Kremer LC, van den Bos C, van der Pal HJ, Heinen RC, et al. Medical assessment of adverse health outcomes in long-term survivors of childhood cancer. Jama. 2007;297:2705–15. 10.1001/jama.297.24.2705 17595271

[4] Gomes C, Geels J, Debray TPA, Malekzadeh A, Asselbergs FW, Linschoten M. Risk prediction models for cancer therapy related cardiac dysfunction in patients with cancer and cancer survivors: systematic review and meta-analysis. Bmj. 2025;390:e084062. 10.1136/bmj-2025-084062 PMC12492976 40987514

[5] Ewer MS, Ewer SM. Cardiotoxicity of anticancer treatments. Nat Rev Cardiol. 2015;12:547–58. 10.1038/nrcardio.2015.65 25962976

[6] Lyon AR, López-Fernández T, Couch LS, Asteggiano R, Aznar MC, Bergler-Klein J, et al. 2022 ESC Guidelines on cardio-oncology developed in collaboration with the European Hematology Association (EHA), the European Society for Therapeutic Radiology and Oncology (ESTRO) and the International Cardio-Oncology Society (IC-OS). Eur Heart J. 2022;43:4229–361. 10.1093/eurheartj/ehac244 36017568

[7] Potts JE, Iliescu CA, Lopez Mattei JC, Martinez SC, Holmvang L, Ludman P, et al. Percutaneous coronary intervention in cancer patients: a report of the prevalence and outcomes in the United States. Eur Heart J. 2019;40:1790–800. 10.1093/eurheartj/ehy769 30500952

[8] Bharadwaj A, Potts J, Mohamed MO, Parwani P, Swamy P, Lopez-Mattei JC, et al. Acute myocardial infarction treatments and outcomes in 6.5 million patients with a current or historical diagnosis of cancer in the USA. Eur Heart J. 2020;41:2183–93. 10.1093/eurheartj/ehz851 31800032

[9] Campia U, Moslehi JJ, Amiri-Kordestani L, Barac A, Beckman JA, Chism DD, et al. Cardio-oncology: vascular and metabolic perspectives: a scientific statement from the American Heart Association. Circulation. 2019;139:e579–602. 10.1161/CIR.0000000000000641 PMC6530491 30786722

[10] Stewart MH, Jahangir E, Polin NM. Valvular Heart Disease in Cancer Patients: Etiology, Diagnosis, and Management. Curr Treat Options Cardiovasc Med. 2017;19:53. 10.1007/s11936-017-0550-6 28547673

[11] Yeh ET, Bickford CL. Cardiovascular complications of cancer therapy: incidence, pathogenesis, diagnosis, and management. J Am Coll Cardiol. 2009;53:2231–47. 10.1016/j.jacc.2009.02.050 19520246

[12] Lyon AR, Dent S, Stanway S, Earl H, Brezden-Masley C, Cohen-Solal A, et al. Baseline cardiovascular risk assessment in cancer patients scheduled to receive cardiotoxic cancer therapies: a position statement and new risk assessment tools from the Cardio-Oncology Study Group of the Heart Failure Association of the European Society of Cardiology in collaboration with the International Cardio-Oncology Society. Eur J Heart Fail. 2020;22:1945–60. 10.1002/ejhf.1920 PMC8019326 32463967

[13] Armenian SH, Lacchetti C, Barac A, Carver J, Constine LS, Denduluri N, et al. Prevention and monitoring of cardiac dysfunction in survivors of adult cancers: American Society of Clinical Oncology Clinical Practice Guideline. J Clin Oncol. 2017;35:893–911. 10.1200/JCO.2016.70.5400 27918725

[14] Feldman DR, Ardeshir-Rouhani-Fard S, Monahan P, Sesso HD, Fung C, Williams AM, et al. Predicting cardiovascular disease among testicular cancer survivors after modern cisplatin-based chemotherapy: application of the Framingham risk score. Clin Genitourin Cancer. 2018;16:e761–9. 10.1016/j.clgc.2018.01.011 PMC6063791 29534941

[15] Altena R, Hubbert L, Kiani NA, Wengström Y, Bergh J, Hedayati E. Evidence-based prediction and prevention of cardiovascular morbidity in adults treated for cancer. Cardiooncology. 2021;7:20. 10.1186/s40959-021-00105-y PMC8161987 34049593

[16] Ezaz G, Long JB, Gross CP, Chen J. Risk prediction model for heart failure and cardiomyopathy after adjuvant trastuzumab therapy for breast cancer. J Am Heart Assoc. 2014;3:e000472. 10.1161/JAHA.113.000472 PMC3959671 24584736

[17] Abdel-Qadir H, Thavendiranathan P, Austin PC, Lee DS, Amir E, Tu JV, et al. Development and validation of a multivariable prediction model for major adverse cardiovascular events after early stage breast cancer: a population-based cohort study. Eur Heart J. 2019;40:3913–20. 10.1093/eurheartj/ehz460 31318428

[18] Abdel-Qadir H, Thavendiranathan P, Austin PC, Lee DS, Amir E, Tu JV, et al. The Risk of Heart Failure and Other Cardiovascular Hospitalizations After Early Stage Breast Cancer: A Matched Cohort Study. J Natl Cancer Inst. 2019;111:854–62. 10.1093/jnci/djy218 PMC6695318 30715404

[19] Chen Y, Chow EJ, Oeffinger KC, Border WL, Leisenring WM, Meacham LR, et al. Traditional cardiovascular risk factors and individual prediction of cardiovascular events in childhood cancer survivors. J Natl Cancer Inst. 2020;112:256–65. 10.1093/jnci/djz108 PMC7073918 31161223

[20] Armenian SH, Armstrong GT, Aune G, Chow EJ, Ehrhardt MJ, Ky B, et al. Cardiovascular Disease in Survivors of Childhood Cancer: Insights Into Epidemiology, Pathophysiology, and Prevention. J Clin Oncol. 2018;36:2135–44. 10.1200/JCO.2017.76.3920 PMC6804893 29874141

[21] Leerink JM, de Baat EC, Feijen EAM, Bellersen L, van Dalen EC, Grotenhuis HB, et al. Cardiac Disease in Childhood Cancer Survivors: Risk Prediction, Prevention, and Surveillance: JACC CardioOncology State-of-the-Art Review. JACC CardioOncol. 2020;2:363–78. 10.1016/j.jaccao.2020.08.006 PMC8352294 34396245

[22] Gallicchio L, Tonorezos E, de Moor JS, Elena J, Farrell M, Green P, et al. Evidence Gaps in Cancer Survivorship Care: A Report From the 2019 National Cancer Institute Cancer Survivorship Workshop. J Natl Cancer Inst. 2021;113:1136–42. 10.1093/jnci/djab049 PMC8418428 33755126

[23] Ellis KR, Koechlin H, Rudaz M, Gerido LH, Hecht HK, Jones C, et al. Predictors and Interdependence of Quality of Life in a Random Sample of Long-Term Young Breast Cancer Survivors and Their Biological Relatives. Cancer Med. 2024;13:e70328. 10.1002/cam4.70328 PMC11519995 39470180

[24] He D, Wang W, Luo X, Wang Y. Cluster analysis of hotspots and research trends of epirubicin-induced cardiotoxicity: a bibliometric study. Front Pharmacol. 2025;16:1616162. 10.3389/fphar.2025.1616162 PMC12405342 40910006

[25] Herrmann J, Lerman A, Sandhu NP, Villarraga HR, Mulvagh SL, Kohli M. Evaluation and management of patients with heart disease and cancer: cardio-oncology. Mayo Clin Proc. 2014;89:1287–306. 10.1016/j.mayocp.2014.05.013 PMC4258909 25192616

[26] Lenneman CG, Sawyer DB. Cardio-Oncology: An Update on Cardiotoxicity of Cancer-Related Treatment. Circ Res. 2016;118:1008–20. 10.1161/CIRCRESAHA.115.303633 26987914

[27] Attar A, Behnagh AK, Hosseini M, Amanollahi F, Shafiekhani P, Kabir A. Beta-Blockers for Primary Prevention of Anthracycline-Induced Cardiac Toxicity: An Updated Meta-Analysis of Randomized Clinical Trials. Cardiovasc Ther. 2022;2022:8367444. 10.1155/2022/8367444 PMC9818032 36687509

[28] Sorin M, Prosty C, Ghaleb L, Nie K, Katergi K, Shahzad MH, et al. Neoadjuvant Chemoimmunotherapy for NSCLC: A Systematic Review and Meta-Analysis. JAMA Oncol. 2024;10:621–33. 10.1001/jamaoncol.2024.0057 PMC10958389 38512301

[29] Darby SC, Cutter DJ, Boerma M, Constine LS, Fajardo LF, Kodama K, et al. Radiation-related heart disease: current knowledge and future prospects. Int J Radiat Oncol Biol Phys. 2010;76:656–65. 10.1016/j.ijrobp.2009.09.064 PMC3910096 20159360

[30] Fang K, Zhang Y, Liu W, He C. Effects of angiotensin-converting enzyme inhibitor/angiotensin receptor blocker use on cancer therapy-related cardiac dysfunction: a meta-analysis of randomized controlled trials. Heart Fail Rev. 2021;26:101–9. 10.1007/s10741-019-09906-x 31900787

[31] Lewinter C, Nielsen TH, Edfors LR, Linde C, Bland JM, LeWinter M, et al. A systematic review and meta-analysis of beta-blockers and renin-angiotensin system inhibitors for preventing left ventricular dysfunction due to anthracyclines or trastuzumab in patients with breast cancer. Eur Heart J. 2022;43:2562–9. 10.1093/eurheartj/ehab843 34951629

[32] Albini A, Pennesi G, Donatelli F, Cammarota R, De Flora S, Noonan DM. Cardiotoxicity of anticancer drugs: the need for cardio-oncology and cardio-oncological prevention. J Natl Cancer Inst. 2010;102:14–25. 10.1093/jnci/djp440 PMC2802286 20007921

[33] Henriksen PA. Anthracycline cardiotoxicity: an update on mechanisms, monitoring and prevention. Heart. 2018;104:971–7. 10.1136/heartjnl-2017-312103 29217634

[34] Armenian SH, Xu L, Ky B, Sun C, Farol LT, Pal SK, et al. Cardiovascular Disease Among Survivors of Adult-Onset Cancer: A Community-Based Retrospective Cohort Study. J Clin Oncol. 2016;34:1122–30. 10.1200/JCO.2015.64.0409 PMC7357493 26834065

[35] Weaver KE, Foraker RE, Alfano CM, Rowland JH, Arora NK, Bellizzi KM, et al. Cardiovascular risk factors among long-term survivors of breast, prostate, colorectal, and gynecologic cancers: a gap in survivorship care? J Cancer Surviv. 2013;7:253–61. 10.1007/s11764-013-0267-9 PMC3756807 23417882

[36] Addison D, Branch M, Baik AH, Fradley MG, Okwuosa T, Reding KW, et al. Equity in Cardio-Oncology Care and Research: A Scientific Statement From the American Heart Association. Circulation. 2023;148:297–308. 10.1161/CIR.0000000000001158 37377045

[37] Murg SI, Matiș L, Moldovan AF, Popovici DI, Negru AG, Ghitea TC, et al. Association Between Advanced TNM Stages and Increased Risk of Cardiac Dysfunction in Patients with LVEF < 50. Medicina (Kaunas). 2025;61(2):301. 10.3390/medicina61020301. PMC11857368 40005419

[38] Deng Z, Jung M, Li M, Li J, Langston ME, Chung BI. Longitudinal patterns of cardiovascular disease and cancer mortality in renal cell carcinoma patients by tumor stage. JACC CardioOncology. 2025;7:627–40. 10.1016/j.jaccao.2025.06.004 PMC12441620 40841112

[39] Kuwabara M, Fukuuchi T, Aoki Y, Mizuta E, Ouchi M, Kurajoh M, et al. Exploring the multifaceted nexus of uric acid and health: a review of recent studies on diverse diseases. Biomolecules. 2023. 10.3390/biom13101519. PMC10604821 37892201

[40] Howard SC, Avagyan A, Workeneh B, Pui CH. Tumour lysis syndrome. Nat Rev Dis Primers. 2024;10:58. 10.1038/s41572-024-00542-w 39174582

[41] Yuan Q, Ye R, Qian Y, Yu H, Zhou Y, Cui X, et al. Machine learning and SHAP value interpretation for predicting the response to neoadjuvant chemotherapy and long-term clinical outcomes in Chinese female breast cancer. Ann Med. 2025;57:2541316. 10.1080/07853890.2025.2541316 PMC12322993 40754951

[42] Wang Y, Pan Z, Li S, Cai H, Huang Y, Zhuang J, et al. Prediction and validation of pathologic complete response for locally advanced rectal cancer under neoadjuvant chemoradiotherapy based on a novel predictor using interpretable machine learning. Eur J Surg Oncol. 2024;50:108738. 10.1016/j.ejso.2024.108738 39395242

[43] Lei J, Zhai J, Zhang Y, Qi J, Sun C. Supervised machine learning models for predicting sepsis-associated liver injury in patients with sepsis: development and validation study based on a multicenter cohort study. J Med Internet Res. 2025;27:e66733. 10.2196/66733 PMC12149780 40418571

[44] Fox ER, Samdarshi TE, Musani SK, Pencina MJ, Sung JH, Bertoni AG, et al. Development and Validation of Risk Prediction Models for Cardiovascular Events in Black Adults: The Jackson Heart Study Cohort. JAMA Cardiol. 2016;1:15–25. 10.1001/jamacardio.2015.0300 PMC5115626 27437649

