# Supplementary material for: Interpretable machine learning for cardiovascular disease risk prediction in cancer survivors: development and internal validation
Source: Cardiooncology. 2026 Jul 20;12:127. doi: 10.1186/s40959-026-00547-2 (PMC13663288; doi:10.1186/s40959-026-00547-2)
Supplement: Supplementary file 1 [file 40959_2026_547_MOESM1_ESM.pdf]

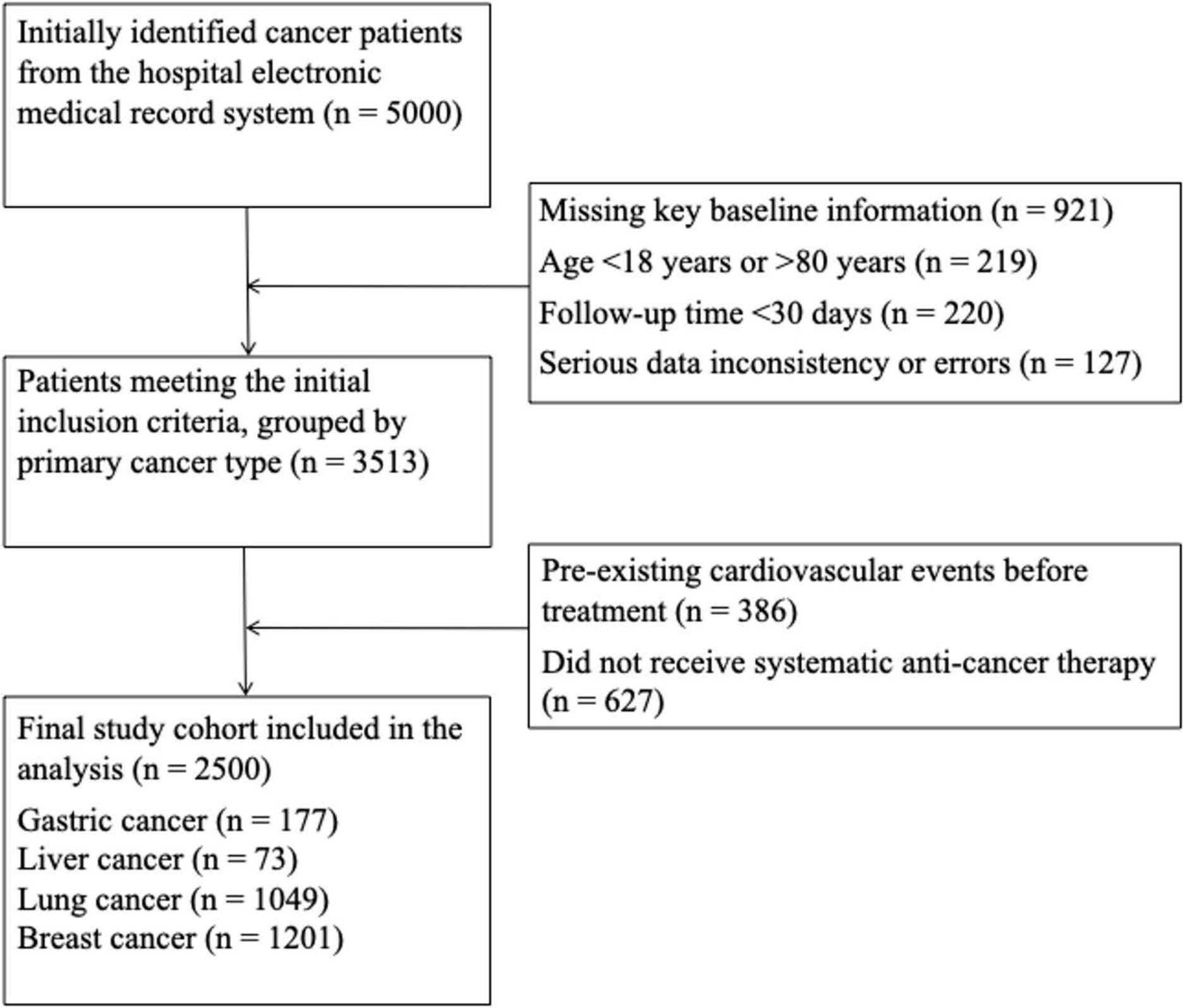
Initially identified cancer patients from the hospital electronic medical record system (n = 5000)
Missing key baseline information (n = 921)
Age <18 years or >80 years (n = 219)
Follow-up time <30 days (n = 220)
Serious data inconsistency or errors (n = 127)
Patients meeting the initial inclusion criteria, grouped by primary cancer type (n = 3513)
Pre-existing cardiovascular events before treatment (n = 386)
Did not receive systematic anti-cancer therapy (n = 627)
Final study cohort included in the analysis (n = 2500)
Gastric cancer (n = 177)
Liver cancer (n = 73)
Lung cancer (n = 1049)
Breast cancer (n = 1201)
