# Supplementary material for: Interpretable machine learning for cardiovascular disease risk prediction in cancer survivors: development and internal validation
Source: Cardiooncology. 2026 Jul 20;12:127. doi: 10.1186/s40959-026-00547-2 (PMC13663288; doi:10.1186/s40959-026-00547-2)
Supplement: Supplementary file 2 [file 40959_2026_547_MOESM2_ESM.pdf]

Univariate Cox Regression Analysis

Hazard Ratios with 95% Confidence Intervals

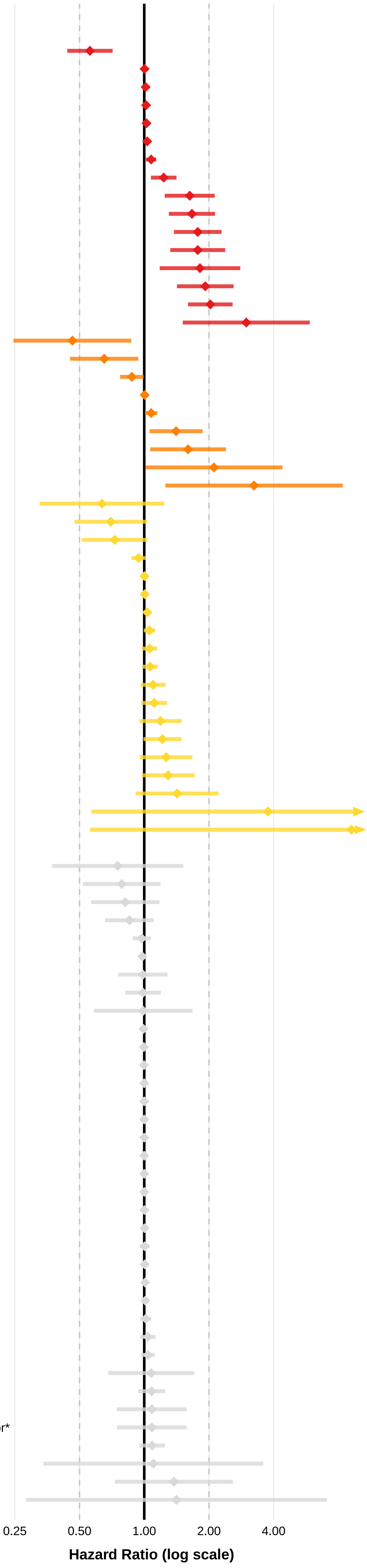

Variable	HR	95% CI	*P* − value
Breast cancer	0.559	(0.438−0.713)	*P* < 0.001
UA	1.003	(1.002−1.005)	*P* < 0.001
Cr	1.015	(1.006−1.024)	*P* < 0.001
Diastolic BP	1.020	(1.005−1.035)	0.007
Systolic BP	1.024	(1.017−1.032)	*P* < 0.001
Age	1.032	(1.019−1.045)	*P* < 0.001
Na	1.076	(1.019−1.137)	0.009
Fibrinogen	1.232	(1.074−1.413)	0.003
Chest radiotherapy*	1.628	(1.245−2.128)	*P* < 0.001
Gender	1.667	(1.302−2.135)	*P* < 0.001
Fluoropyrimidine*	1.774	(1.374−2.291)	*P* < 0.001
Platin*	1.774	(1.322−2.381)	*P* < 0.001
Chemotherapy*	1.816	(1.180−2.797)	0.007
Hypertension	1.923	(1.419−2.605)	*P* < 0.001
Lung cancer	2.030	(1.599−2.577)	*P* < 0.001
Cys-C	2.983	(1.511−5.891)	0.002
Gastric cancer	0.463	(0.246−0.871)	0.017
EGFR-TKI*	0.651	(0.452−0.939)	0.022
MPV	0.876	(0.772−0.993)	0.039
ALP	1.004	(1.001−1.008)	0.012
Eosin%	1.077	(1.007−1.151)	0.030
Taxane*	1.407	(1.059−1.869)	0.019
Anti-HER2*	1.599	(1.066−2.398)	0.023
Mono	2.110	(1.012−4.400)	0.046
Eosin	3.241	(1.253−8.384)	0.015
Other targeted therapy*	0.636	(0.326−1.241)	0.184
Immunosuppressant*	0.699	(0.475−1.030)	0.070
K	0.729	(0.513−1.035)	0.077
DBIL	0.942	(0.872−1.017)	0.128
GGT	1.003	(0.999−1.006)	0.101
Hb	1.005	(0.997−1.014)	0.193
Cl	1.033	(0.993−1.075)	0.110
WBC	1.058	(0.995−1.124)	0.071
Glucose	1.058	(0.973−1.149)	0.185
Urea	1.064	(0.979−1.156)	0.145
TNM stage	1.099	(0.962−1.256)	0.164
T stage	1.112	(0.969−1.277)	0.129
PDW	1.191	(0.950−1.493)	0.130
Lymph	1.216	(0.992−1.491)	0.060
RBC	1.264	(0.954−1.675)	0.102
Other chemotherapy*	1.293	(0.974−1.716)	0.076
Diabetes	1.421	(0.911−2.217)	0.121
Mg	3.764	(0.571−24.819)	0.168
Hct	9.808	(0.565−170.221)	0.117
Baso	63.450	(0.114−35265.302)	0.198
P	0.752	(0.372−1.520)	0.428
Antiangiogenic-TKI*	0.786	(0.519−1.190)	0.255
Anthracycline*	0.816	(0.565−1.177)	0.277
Targeted therapy*	0.853	(0.658−1.107)	0.232
TT	0.974	(0.884−1.075)	0.605
ALB	0.980	(0.948−1.013)	0.233
Radiotherapy*	0.986	(0.758−1.284)	0.919
M stage	0.988	(0.816−1.195)	0.898
ALB/GLO ratio	0.989	(0.583−1.676)	0.966
TP	0.991	(0.970−1.013)	0.436
Neut%	0.996	(0.983−1.009)	0.552
MCHC	0.997	(0.985−1.009)	0.655
AST	0.999	(0.988−1.010)	0.887
PTA	1.000	(0.989−1.011)	0.944
ALT	1.000	(0.992−1.008)	0.962
PLT	1.000	(0.998−1.002)	0.878
ChE	1.000	(1.000−1.000)	0.733
GLO	1.000	(0.971−1.030)	0.992
Lymph%	1.000	(0.986−1.015)	0.950
TBIL	1.002	(0.980−1.024)	0.888
MCV	1.004	(0.984−1.024)	0.706
MCH	1.004	(0.952−1.059)	0.879
TBA	1.005	(0.971−1.041)	0.761
IBIL	1.009	(0.983−1.035)	0.499
APTT	1.011	(0.974−1.050)	0.562
Mono%	1.016	(0.956−1.080)	0.604
RDW-CV	1.039	(0.957−1.129)	0.356
Neut	1.040	(0.966−1.120)	0.294
Baso%	1.077	(0.679−1.709)	0.751
PT	1.083	(0.938−1.250)	0.278
Immunotherapy*	1.085	(0.747−1.577)	0.669
Immune checkpoint inhibitor*	1.085	(0.747−1.577)	0.669
N stage	1.088	(0.949−1.247)	0.227
Ca	1.103	(0.340−3.582)	0.870
Liver cancer	1.374	(0.731−2.583)	0.324
PT-INR	1.412	(0.282−7.078)	0.675

Significance Level — *P* < 0.01 — *P* < 0.05 — *P* < 0.20 — *P* ≥ 0.20

* Adjusted for cancer type
