# Supplementary material for: Interpretable machine learning for cardiovascular disease risk prediction in cancer survivors: development and internal validation
Source: Cardiooncology. 2026 Jul 20;12:127. doi: 10.1186/s40959-026-00547-2 (PMC13663288; doi:10.1186/s40959-026-00547-2)

Spearman Correlation Matrix of Clinical Variables

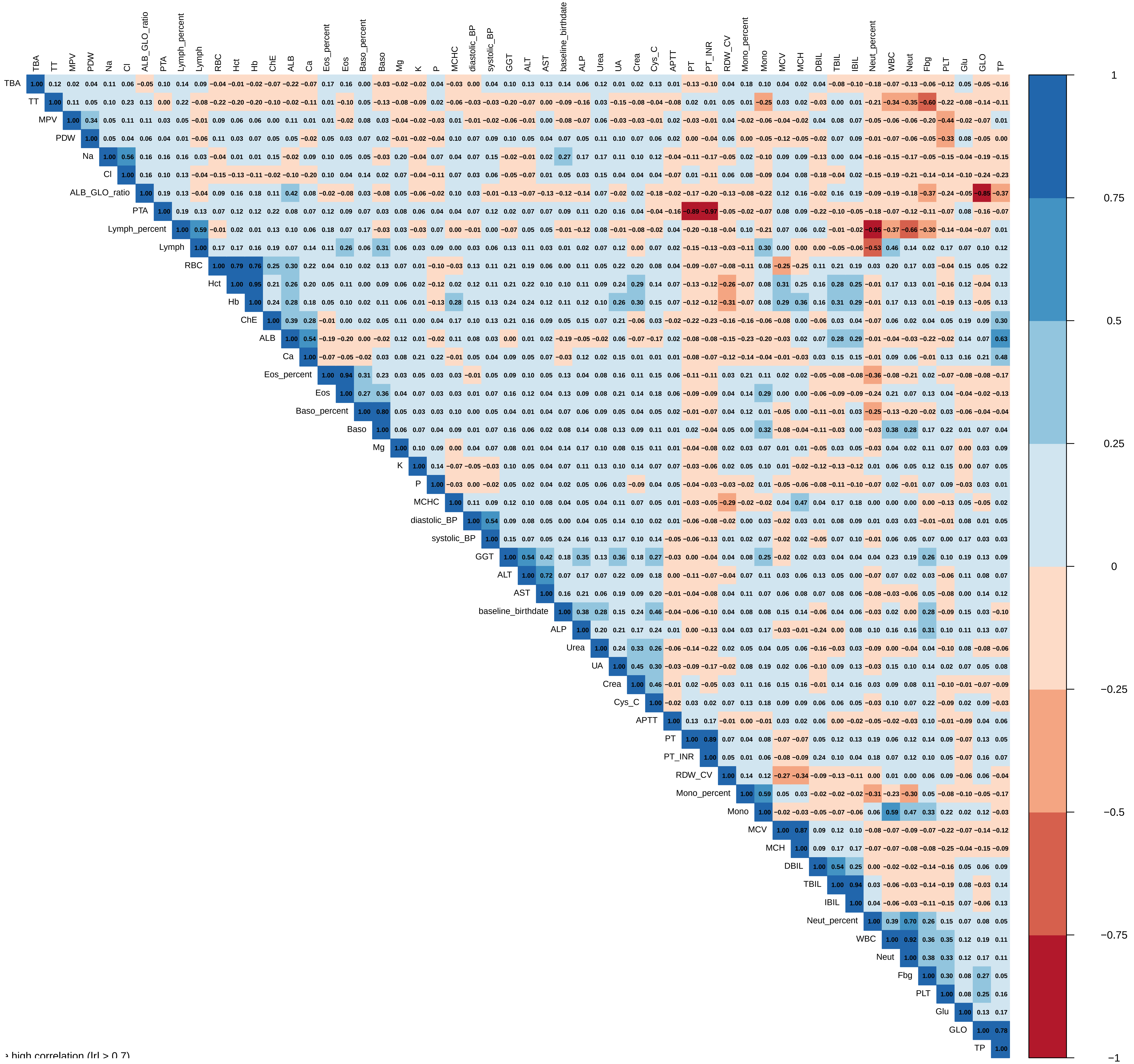

Supplement: Supplementary file 3 [file 40959_2026_547_MOESM3_ESM.pdf]
