# Supplementary material for: Interpretable machine learning for cardiovascular disease risk prediction in cancer survivors: development and internal validation
Source: Cardiooncology. 2026 Jul 20;12:127. doi: 10.1186/s40959-026-00547-2 (PMC13663288; doi:10.1186/s40959-026-00547-2)
Supplement: Supplementary file 4 [file 40959_2026_547_MOESM4_ESM.pdf]

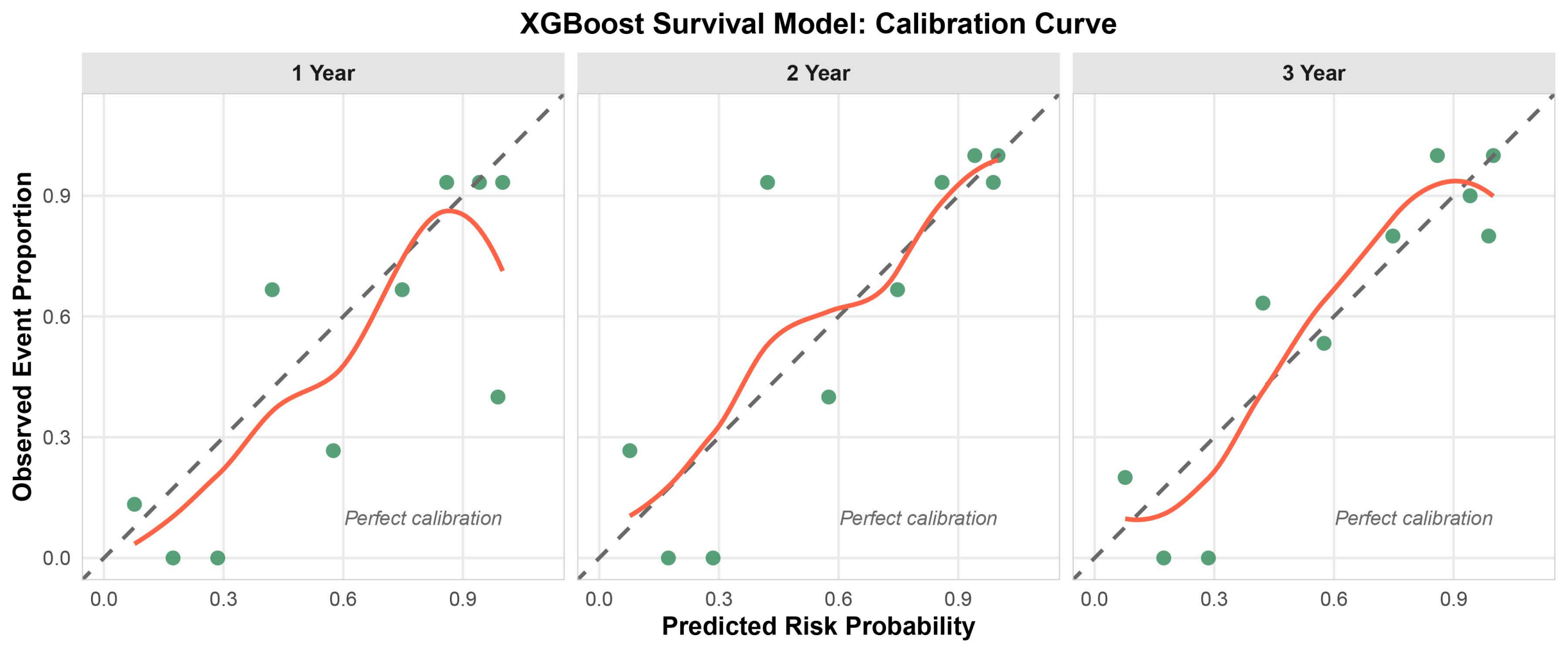
XGBoost Survival Model: Calibration Curve
1 Year
2 Year
3 Year
Observed Event Proportion
0.0
0.3
0.6
0.9
Perfect calibration
Perfect calibration
Perfect calibration
0.0
0.3
0.6
0.9
0.0
0.3
0.6
0.9
0.0
0.3
0.6
0.9
Predicted Risk Probability
