# Supplementary material for: Interpretable machine learning for cardiovascular disease risk prediction in cancer survivors: development and internal validation
Source: Cardiooncology. 2026 Jul 20;12:127. doi: 10.1186/s40959-026-00547-2 (PMC13663288; doi:10.1186/s40959-026-00547-2)
Supplement: Supplementary file 5 [file 40959_2026_547_MOESM5_ESM.pdf]

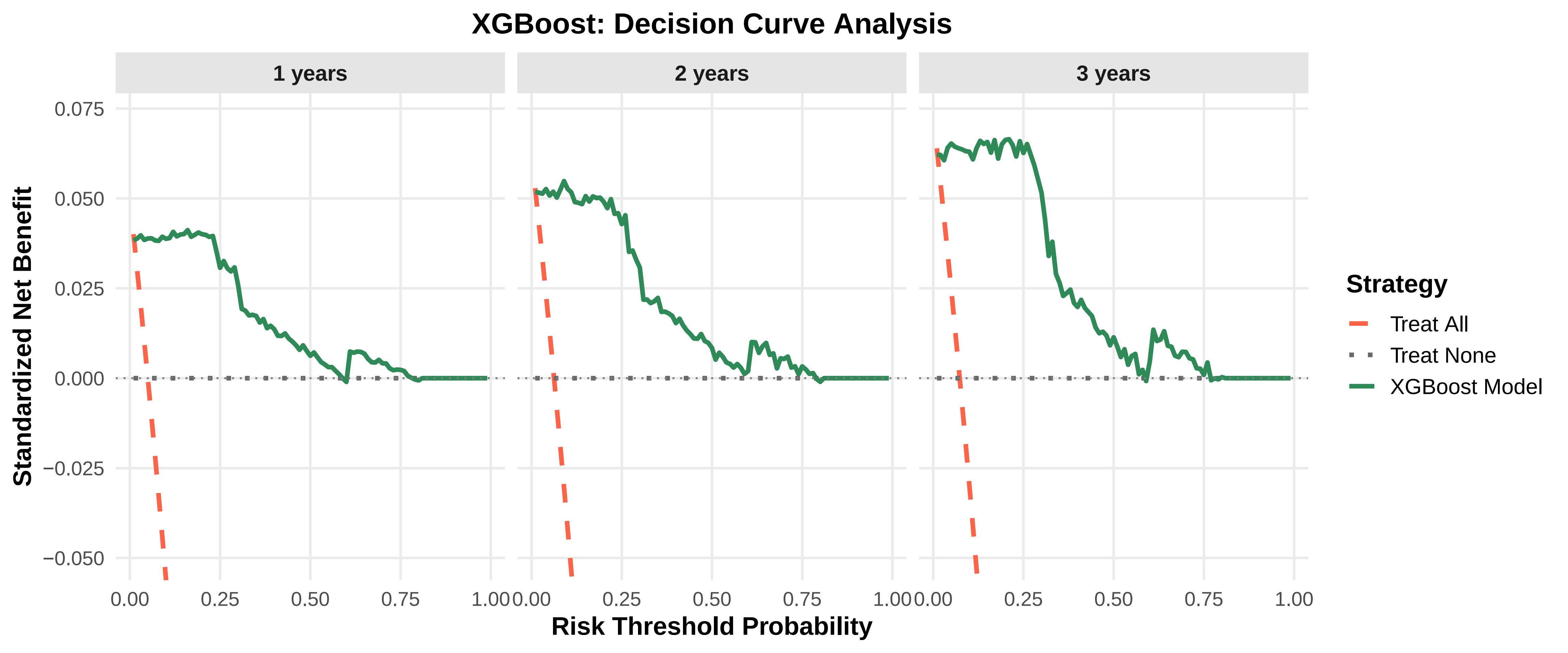
XGBoost: Decision Curve Analysis
1 years
2 years
3 years
Standardized Net Benefit
0.075
0.050
0.025
0.000
−0.025
−0.050
0.00
0.25
0.50
0.75
1.00
Risk Threshold Probability
Strategy
Treat All
Treat None
XGBoost Model
