# Supplementary material for: Interpretable machine learning for cardiovascular disease risk prediction in cancer survivors: development and internal validation
Source: Cardiooncology. 2026 Jul 20;12:127. doi: 10.1186/s40959-026-00547-2 (PMC13663288; doi:10.1186/s40959-026-00547-2)
Supplement: Supplementary file 6 [file 40959_2026_547_MOESM6_ESM.pdf]

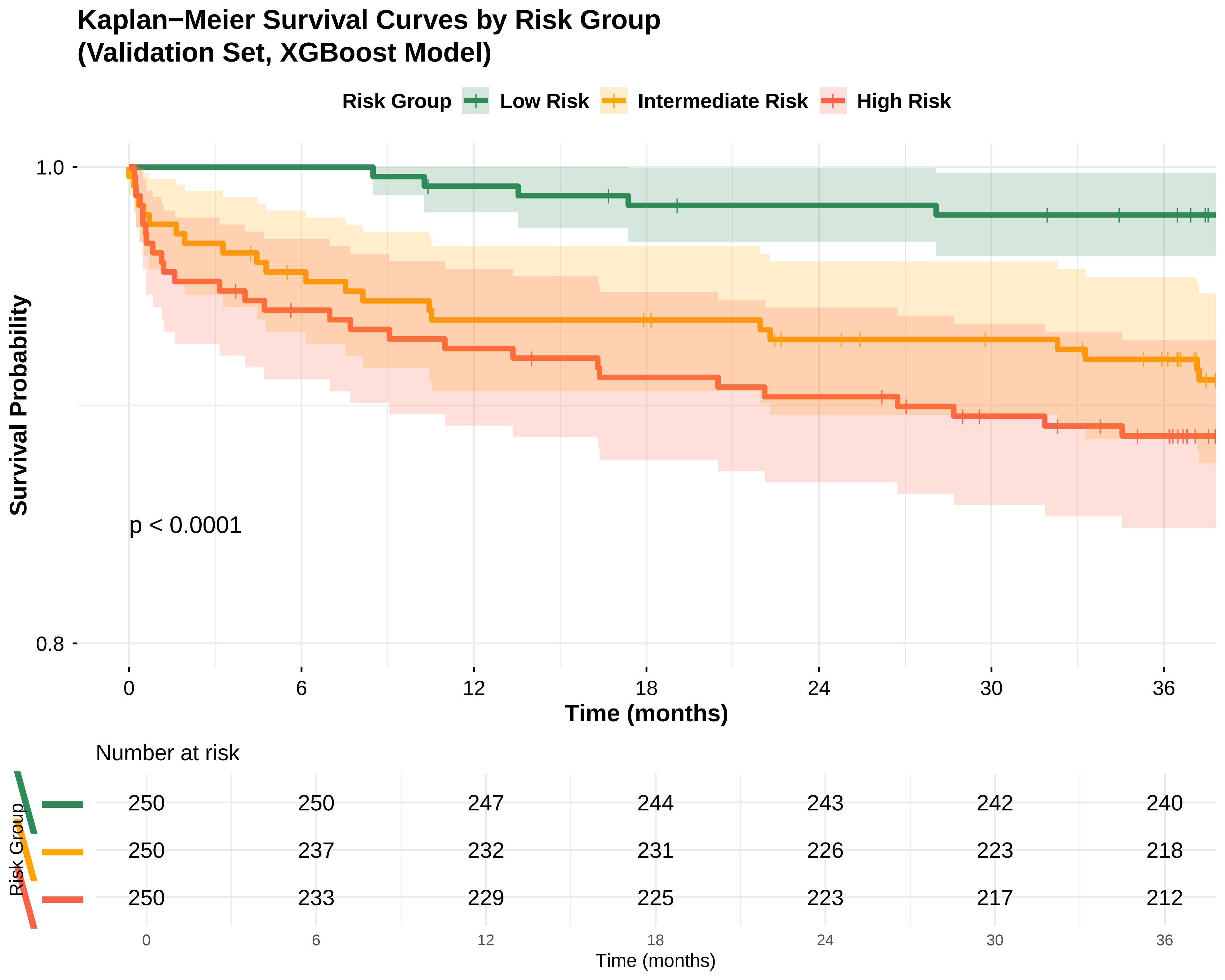

Kaplan−Meier Survival Curves by Risk Group
(Validation Set, XGBoost Model)
Risk Group
Low Risk
Intermediate Risk
High Risk
Survival Probability
1.0
0.8
p < 0.0001
0
6
12
18
24
30
36
Time (months)
Number at risk
Risk Group
250 250 247 244 243 242 240
250 237 232 231 226 223 218
250 233 229 225 223 217 212
0
6
12
18
24
30
36
Time (months)
